# Proteomic Selection of Immunodiagnostic Antigens for *Trypanosoma congolense*

**DOI:** 10.1371/journal.pntd.0002936

**Published:** 2014-06-12

**Authors:** Jennifer R. Fleming, Lalitha Sastry, Thomas W. M. Crozier, Grant B. Napier, Lauren Sullivan, Michael A. J. Ferguson

**Affiliations:** 1 Division of Biological Chemistry and Drug Discovery, College of Life Sciences, University of Dundee, Dundee, United Kingdom; 2 Global Alliance for Livestock Veterinary Medicines, Doherty Building, Pentlands Science Park, Edinburgh, United Kingdom; McGill university, Canada

## Abstract

Animal African Trypanosomosis (AAT) presents a severe problem for agricultural development in sub-Saharan Africa. It is caused by several trypanosome species and current means of diagnosis are expensive and impractical for field use. Our aim was to discover antigens for the detection of antibodies to *Trypanosoma congolense*, one of the main causative agents of AAT. We took a proteomic approach to identify potential immunodiagnostic parasite protein antigens. One hundred and thirteen proteins were identified which were selectively recognized by infected cattle sera. These were assessed for likelihood of recombinant protein expression in *E. coli* and fifteen were successfully expressed and assessed for their immunodiagnostic potential by ELISA using pooled pre- and post-infection cattle sera. Three proteins, members of the invariant surface glycoprotein (ISG) family, performed favorably and were then assessed using individual cattle sera. One antigen, Tc38630, evaluated blind with 77 randomized cattle sera in an ELISA assay gave sensitivity and specificity performances of 87.2% and 97.4%, respectively. Cattle immunoreactivity to this antigen diminished significantly following drug-cure, a feature helpful for monitoring the efficacy of drug treatment.

## Author Summary

Animal African Trypanosomosis (AAT) is a set of diseases whereby animals are infected with single-cell parasites that replicate in their bloodstream. The disease in cattle results in weight-loss and death, and AAT is a significant veterinary problem for sub-Saharan Africa. One of the principal trypanosome species responsible for AAT in cattle is *Trypanosoma congolense* and, although there are drug-treatments for these infections, current diagnostic methods are impractical for field use. Our aim was to discover protein molecules from the parasite to which infected animals make antibodies, to then make these proteins in bacteria and to subsequently demonstrate that they can be used to detect antibodies in cattle serum, thus diagnosing AAT. To discover the diagnostic proteins, we dissolved parasites in a detergent solution and applied them to beads coated with antibodies from infected cattle and to beads coated with antibodies from un-infected cattle. We then compared the proteins bound to each and selected those proteins that were at least 100-fold enriched by the infected cattle antibodies. We refined this list, according to practical and performance considerations, and settled on one protein, called Tc38630. Testing Tc38630 with cattle sera showed that it can detect about nine out of ten AAT infections.

## Introduction

Animal African Trypanosomosis (AAT) presents a severe problem for agricultural development in sub-Saharan Africa. The economic loss in cattle production is estimated to be between US$ 1 billion per annum [1]–[3], increasing to US$ 5 billion when secondary costs are taken into consideration [1], [3], [4]. It is estimated that around 60 million cattle are at risk in endemic regions [1], [4]. In addition, AAT affects many other domestic livestock such as pigs, camels, goats, sheep and horses. There are no vaccines and treatment is usually via intramuscular administration using trypanocides, either diminazene (therapeutically) or isometamidium (prophylactically). Current diagnostics methods are laborious (microscopy), expensive (PCR) and generally impractical for use in the field, at the point of treatment.

AAT is caused by several species of protozoan parasites of the genus trypanosoma, including *T. congolense*, *T. brucei*, *T. vivax* and *T. evansi*. [1]. *T. congolense* is the most pathogenic and main causative agent of cattle AAT and is transmitted in Africa by tsetse flies of the genus Glossina [1], [5]. Symptoms of *T. congolense* AAT include anaemia, weakness, weight loss and, in most untreated cases, death [5], [6]. These symptoms are often used to clinically diagnose AAT, although they are congruent with many other anaemia causing diseases prevalent in the endemic regions including babesiosis, anaplasmosis, hemonchosis, and theileriosis. Wrong diagnosis is costly and counter productive to efficient treatment.

Currently, specific diagnostics rely on microscopic detection of the parasites, the laboratory detection of specific antibodies or the detection of parasite DNA by PCR [5], [6]. A card agglutination test (CATT), such as that used to detect human African trypanosomosis in the field, is available for *T. evansi* infections [5] but is not applicable to *T. congolense* and *T. vivax* infections. Diagnosis in the field for these pathogens currently relies on whole cell lysate tests that suffer from antigen instability, reproducibility and specificity problems [5].

With this in mind, we set out to discover new diagnostic antigens for *T. congolense* that might be compatible with ELISA assays and subsequent development into lateral flow devices.

In order to identify antigens for recombinant production and test development, we utilised a proteomic approach similar to that recently used to identify invariant surface glycoprotein (ISG) 65 as a potential diagnostic antigen for human African trypanosomosis [7]. Briefly, this method involves loading identical amounts of parasite whole-cell detergent lysates onto identical amounts of immobilized IgG isolated from the same animals before and after experimental infection and then comparing, by label-free quantitative proteomics, the proteins subsequently eluted from the immobilized IgGs and selecting those >100-fold more highly represented in the eluate from the immobilized infection IgG. This is a systematic approach to antigen identification, as was the identification of *T. vivax* GM6 from a cDNA expression library screen with infected bovine sera [8], [9]. Both are alternatives to candidate-based approaches, whereby potential diagnostic antigens are selected based on literature precedent. A successful example of the latter for AAT is the selection of HSP70/BiP [10] because, despite substantial conservation of sequence between species, HSP70/BiP is known to be highly immunogenic.

Here, using the systematic proteomics approach, we report the identification, production and evaluation of several new potential diagnostic antigens. The two best performing antigens were both ISG family members, one of which (Tc38630) has been recently reported as a potential diagnostic antigen for *T. congolense* infections using mouse sera [11]. In this study, we extensively characterise a recombinant Tc38630 construct against diagnostically relevant cattle sera and demonstrated its potential for the diagnosis of *T. congolense* infections in bovines. We also demonstrate its ability to distinguish, to some degree, between successful and failed drug treatments.

## Materials and Methods

### Ethics statement

Rodents were used to propagate sufficient *T. congolense* parasites to make the detergent lysates for immunoaffinity chromatography and proteomics. The animal procedures were carried out according the United Kingdom Animals (Scientific Procedures) Act 1986 and according to specific protocols approved by The University of Dundee Ethics Committee and as defined and approved in the UK Home Office Project License PPL 60/3836 held by MAJF.

All cattle studies were reviewed by the ClinVet Animal Ethics Committee (CAEC), who authorised the clinical research organization to conduct the studies (approval numbers CV12/884; CV12/928 and CV12/885). The study protocols were designed to allow the use of the study animals in compliance with the ClinVet Policy on the ethical use of animals and the South African National Standard “SANS 10386:2008 The care and use of animals for scientific purposes”. Permission to do research was granted by the South African Department of Agriculture, Forestry and Fisheries (DAFF) under Section 20 of the South African Animal Diseases Act, 1984 (ACT NO. 35 of 1984).

### Cattle sera from *T. congolense* infected and uninfected animals

Cattle sera were obtained from experimental infections being conducted as part of exploratory clinical efficacy studies, using preliminary formulations of novel trypanocides, at ClinVet International (Pty) Ltd and provided by GALVmed. All animals were obtained from tsetse free areas and shown to below the limits of trypanosomosis detection by polymerase chain reaction–restriction fragment length polymorphism (PCR-RFLP) assay [12], performed by the Molecular Diagnostic Services laboratory, Department of Veterinary Tropical Diseases, Pretoria. Animals were maintained in fly-proof facilities in a tsetse free area (ClinVet International (Pty) Ltd, Bloemfontein, South Africa). Serum samples for IgG purification were collected at either 7 days pre-infection or 28 days post infection with *T. congolense* strains 02J and 31J from Centro de Biotecnologia, Faculdade de Veterinária, Universidade Eduardo Mondlane, Maputo, Mozambique and strains KONT2/133 and KONT2/151, isolated in 2004 from naturally infected cattle in Kontcha, Cameroon and obtained from the Institute of Tropical Medicine, Antwerp, Belgium. Each animal was only infected with one strain.

Test sera were obtained from a total of forty calves from Clinvet studies that were collected at weekly intervals until 98 days post-infection (dpi). These animals were subjected to drug treatments on dpi 10 or 35. Sera from −1, 0, 7, 14, 28, 35, 42, 49, 63, 77, 91 and 98 dpi were evaluated by ELISA. A panel of 77 randomised and blinded (at source) *T. congolense* sera were also evaluated against the best candidate antigen from this study. Parasitaemia levels were monitored by microscopy and packed cell volume (PCV) by standard methods [13].

### IgG purification from serum

Aliquots (50 µl) of sera each from 13 post-infected (day +28) and the same 13 pre-infected (day −7) calves were pooled separately. Each pool was applied to a 1 ml protein G column (GE Healthcare) equilibrated in phosphate buffered saline (PBS). The columns were washed with 10 ml of PBS and the bound IgG antibodies were eluted with 50 mM sodium citrate pH 2.8, and collected in 1 ml fractions into tubes containing 200 µl of 1 M Tris-HCl, buffer pH 8.5. Peak fractions containing IgG were combined and dialysed for 16 h against coupling buffer (0.1 M NaHCO_3_, 0.5 M NaCl, pH 8.3). The purified IgGs were then coupled at 4 mgmL^−1^ of packed gel to CNBr-activated Sepharose 4B beads following the procedures detailed in [7].

### Preparation *T. congolense* lysate

Three BalbC mice were injected with one stabilate of *T. congolense* strain IL 3000. After five days, mouse blood was harvested with citrate anticoagulant, adjusted to 5×10^4^ parasites per ml with PBS and aliquots of 0.2 ml (1×10^4^ mL^−1^ parasites) were injected into the peritoneal cavity of 44 NMR1 mice. The mouse blood was harvested after 7 days with citrate anticoagulant and centrifuged at 1000× *g* for 10 min at 4°C. Plasma was removed and the buffy layer was resuspended in separation buffer plus glucose (SB+glucose; 57 mM Na_2_HPO_4_, 3 mM KH_2_PO_4_, 44 mM NaCl, 10 gL^−1^ glucose) and applied to a DE52 DEAE-cellulose (Whatman) column that had been pre-equilibrated with SB+glucose. The trypanosomes were washed through the column with SB+glucose, counted, centrifuged (900 g, 15 min, 4°C), resuspended in 1 ml PBS and then adjusted to 3.7×10^7^ parasites mL^−1^ in 20 ml ice-cold lysis buffer (50 mM Na_2_PO_4_, pH 7.2, 2% *n*-octyl β-D-glucopyranoside (nOG) detergent, 1 mM PMSF, 1 mM TLCK, 1 µg mL^−1^ aprotinin, 1 µg mL^−1^ leupeptin and 1× Roche protease cocktail minus EDTA). The lysate was incubated for 30 min on ice and then centrifuged at 100,000 *g* for 1 h at 4°C.

### Immunoprecipitation

Identical aliquots of *T. congolense* lysate (7×10^8^ cell equivalents) were incubated in parallel with 0.5 ml packed volume of each of the CNBr Sepharose-IgG (post-infection and pre-infection) gels, rotating for 3 h at 4°C. The gels were then packed into disposable 10 ml columns and washed with 10 ml of 10 mM Na_2_PO_4_, pH 7.2, 200 mM NaCl, 1% nOG, followed by 10 ml of 5 mM Na_2_PO_4_ pH 7.2, 1% nOG. The trypanosome proteins were eluted with 500 µl of 50 mM sodium citrate, pH 2.8, 1% nOG into tubes containing 100 µl of 1 M Tris pH 8.5 for neutralization. The eluates were further concentrated to 270 µl using a centrifugal concentrator (Millipore, 0.5 ml capacity with 10 kDa MW cut off membrane). The concentrates containing the trypanosome proteins were then transferred to low binding Eppendorf tubes and the proteins precipitated by adding 1 ml ice-cold ethanol and incubation for 24 h at −20°C.

### Proteomic protein identification

Following ethanol precipitation, the proteins eluted from the post-infection IgG and pre-infection IgG columns were dissolved in SDS sample buffer, reduced with DTT and run on a precast 4–12% Bis-Tris gradient SDS-PAGE (Invitrogen) using the MES running system. The gel was stained with colloidal Coomassie blue and equivalent regions of the infection and control lanes were cut out, reduced and alkylated with iodoacetamide and digested in-gel with trypsin. The tryptic peptides were analysed by LC-MS/MS on a Thermo Orbitrap XL system and MaxQuant software was used to match peptides to the predicted trypanosome protein databases (combined GeneDB and UniProt predicted protein sequences).

### Data analysis

The program MaxQuant was used to obtain relative intensity data of the peptides recovered from the post-infection and pre-infection (control) IgG columns. This produced a list of 98 proteins found uniquely in the eluate of the infection IgG column ranked by relative intensity (Table S1 in Text S1). A second list of proteins with a >100-fold higher abundance in the post-infection eluate than the pre-infection eluate (15 proteins) was also selected (Table S1 in Text S1). Proteins with peptide coverage under 10% were discarded.

TriTrypDB [14] was used to obtain the predicted protein sequences and names of the selected proteins. Most were annotated as “hypothetical proteins”, in these cases if a trypanosomatid homologue was annotated this name was used. The list was visually inspected and ubiquitin, ribosomes and large structural proteins such as those from the paraflagellar rod and clathrin were removed.

The sequences were further examined against different NCBI databases in order to prioritise targets for recombinant production. The closest homologues (from any organism, trypanosomatid, bovine and humans) were noted. Any close homologues that had produced crystal structures and/or had domains identified by Conserved Domain Database (CDD) [15] were also noted.

A shortlist of proteins was chosen for expression and purification trials based on uniqueness to *T. congolense* (<33% identity to human or bovine homologs) and perceived ease of expression in *E. coli* [16]. Several of these proteins appear to be unique to *T. congolense*, with no close homologues in *T. brucei* or other trypanosomatids. Proteins with homologues in the PDB were considered favourable for expression, as were proteins less than 50 kDa. These were checked using XtalPred [16] for major areas of disorder, coiled coils, signal peptides and transmembrane domains. This produced a list of 15 potential diagnostic protein antigens for expression trials (Table S1 in Text S1).

A pilot IP was initially preformed (data not shown) which produced a shortlist of 14 proteins uniquely recognised by the post-infection IgGs. Twelve of these were selectively recognised by the post-infected IgG eluant in the second IP described here. However, from this list only two proteins, TcIL3000.0.38630 and TcIL3000.2.1660 passed the infection∶control ratio cutoff (>100-fold higher abundance in the post-infection eluate than the pre-infection eluate). Three additional proteins were produced solubly and are described here. However, their infection∶control ratios were low with only 2–4 times enrichment in the infected IgG eluant.

In total, seven proteins could be produced as soluble proteins in sufficient quantities for assessment.

### Cloning

Most of the genes listed in Table 1 were amplified by PCR, using the primers described, from *T. congolense* genomic DNA (kindly provided by Mark Carrington, University of Cambridge) and ligated into pCR2.1-TOPO using the TOPO TA Cloning Kit (Invitrogen). The exception was the gene encoding Tc38630, which was made as a synthetic gene by GenScript in a pUC vector with restriction sites (*Nde*1 and *Xho*1) in place for downstream cloning. The coding sequence was optimized to avoid rare codon combinations in *E. coli* and disfavoured mRNA structures and *cis* elements for protein expression. All of the coding sequences were inserted into a pET15b-derived plasmid (Novagen) modified to include a tobacco etch virus (TEV) protease cleavage site between the His_6_ tag and the coding sequence. Genes for Tc38630, Tc29290 and Tc51750 were also inserted into a modified pET15b-derived plasmid encoding for a TEV cleavable histidine tagged maltose binding protein (kind gift of Dr. Thomas Eadsforth). Recombinant protein expression was achieved with *E. coli* BL21-CodonPlus (DE3) RIPL cells (Stratagene) in autoinduction medium containing 50 µg mL^−1^ ampicillin and 12 µg mL^−1^ chloramphenicol, with the exception of Tc38630 constructs which were expressed in BL21 Gold (DE3) cells in the presence of 50 µg mL^−1^ ampicillin.

**Table 1:** The proteins expressed in this study, their TriTrypDB gene identifiers, the residue ranges expressed and the primers used to PCR amplify them from *T. congolense* genomic DNA.

Protein ID	TriTrypDB gene ID	Residue range	Forward primer (5′ to 3′)	Reverse primer (5′ to 3′)
Tc38630	TcIL3000.0.38630	30–355	N/A	N/A
Tc2530	TcIL3000.9.2530	1–346 (full length)	**C A T A T G** A G G C G A G G G C G T G T	**G G A T C C** T T A C A T T G A C T C A C G C A A C T C A G C
Tc5750	TcIL3000.10.5750	1–311 (full length)	**C A T A T G** T C A A C A A C A G A T A A A T T T C C G	**C T C G A G** T C A A G C C G C A G A T G G A C C
Tc3710	TcIL3000.7.371	1–142 (full length)	**C A T A T G** T C C A G T T G T A T A T T C T G C A G G	**C T C G A G** T T A C A G C A C C T T C T C C A A T G C
Tc29290	TcIL3000.0.29290	26–362	**C A T A T G** G C T G G C A A T T C T A A T G G T G T C	**C T C G A G** T T A G C T C T C C T T T G A A T G G G C A
Tc51750	TcIL3000.0.51750	36–364	**C A T A T G** C T A G A C A G G T T C G G T G C	**C T C G A G** T T A C C C C T T T G G A G T C T C A G T A C G
Tc03060	TcIL3000.0.03060	28–374	**C T C G A G** A C C G A A G A A G C G A A A A C A T T T	**G G A T C C** T T A T T T T G C C A T C A C A A A G A G C C

Bold letters are encode the restriction sites.

### Expression and purification of recombinant protein targets

Cells were grown for 24 h at room temperature, harvested by centrifugation (3,500× *g*, 30 min, 4°C) and lysed with a French press in buffer A (50 mM Tris-HCl, pH 7.5, 250 mM NaCl). Cell debris was removed by centrifugation at 25,000× *g* (30 min, 4°C). The proteins were captured using nickel affinity chromatography on a 5 ml HisTrap column (GE Healthcare) and eluted using an imidazole gradient. This was followed by dialysis into buffer A and proteolytic cleavage of the histidine tag with 1 mg TEV protease per 20 mg protein (4°C, 16 h). The TEV protease carries a histidine tag and so the application of the sample to another HisTrap column removed the protease, any uncleaved protein and the histidine tag itself from the cleaved product, which passed through the column. Size exclusion chromatography on a Superdex 200 26/60 column provided the final stage in purification. With the exception of Tc3710, a final polishing step was performed on a Superose 12 10/30 column. All proteins were >95% pure, as judged by sodium dodecyl sulphate polyacrylamide gel electrophoresis (SDS PAGE) and Coomassie blue staining (Figure S1 in Text S1). The amino acid sequences of the successfully expressed protein domains are shown in (Table S2 in Text S1).

### Enzyme Linked Immunosorbent Assay (ELISA)

White polystyrene Costar untreated plates were coated with 50 µl per well of target protein at a concentration of 2 µg mL^−1^ in plating buffer (0.05 M NaHCO_3_, pH 9.6) for 3 h at room temperature. The plates were then treated overnight at 4°C with PBS containing 5% bovine serum albumin (BSA) and 0.1% Tween-20 to block non-specific adsorption sites. Calf sera were serially diluted from 1∶500 to 1∶16000 and transferred in triplicate by a liquid handling device (Bio-Tek, Precision) to the ELISA plates and incubated for 1 h at room temperature after which they were aspirated and the wells washed with PBS containing 0.1% BSA using the liquid handling device. This wash cycle was repeated 5 times. Biotinylated anti-bovine-IgG (Jackson labs) was added at a dilution of 1∶4000 and incubated for 1 h. Excess anti-bovine-IgG antibody was washed away (as described before) and 50 µl per well of ExtrAvidin-Horse Radish Peroxidase (HRP) at a dilution of 1∶4000 was added to the plates and incubated for 1 h. The solution was aspirated and the wash steps were repeated. Finally, chemiluminescent Femto substrate (Pierce) diluted 1∶5 (*i.e.*, 0.5 ml solution A, 0.5 ml solution B with 4 ml PBS) was applied to the wells at 50 µl per well and plates were read using an Envision plate reader after 2.5 min incubation at 22°C.

### Data analysis and statistics

Bar graphs were generated using Microsoft Excel. Box plots and Receiver Operator Characteristic (ROC) curves were generated using IBM SPSS software. Wilcoxon's signed rank test was used to establish the statistical significance of the antibody response on 28 days post infection versus that on each of the post treatment values.

## Results

### Proteomics approach to diagnostic antigen selection

A proteomics approach was used to identify proteins selectively recognised by the IgG fraction of pooled sera from *T. congolense* infected calves, with the IgG fraction of pooled sera obtained from the same calves prior to infection used as the control. This was achieved by following previously established methods [7]. Briefly, IgG from pooled pre- and 28 day post-infected cattle sera were immobilised onto Sepharose beads and incubated with *T. congolense* lysate. The bound and subsequently eluted proteins were separated by SDS-PAGE. Gel slices were subjected to S-alkylation and tryptic digestion and the tryptic peptides were analysed by LC-MS/MS. These peptides were matched to proteins in the *T. congolense* database and relative intensities assigned using MaxQuant software. This process identified 113 proteins that were either unique or >100-fold more abundant in the eluate from the infection IgGs compared to the eluate from the pre-infection IgGs (Table S1 in Text S1). A large percentage of the proteins thus identified were ‘hypothetical’. The proteins were therefore tentatively assigned to protein family groups based on homology to other annotated trypanosomatid sequences and/or protein fold identification from the CCD [16]. The identified proteins (Table S1 in Text S1) are mostly surface glycoproteins such as variant surface glycoprotein B (VSG_B), expression site associated genes (ESAGs), invariant surface glycoproteins (ISGs), trans-sialidases, unknown proteins that have signal peptides and/or transmembrane domains and proteins involved in trafficking such as soluble NSF attachment protein receptors (SNAREs). These surface and secretory pathway glycoproteins are similar to the proteins identified in a comparable study using human *Trypanosoma brucei gambiense* infection and control IgGs [7]. Fifteen of the identified *T. congolense* proteins were selected for expression trials based on assessment for predicted ease of expression in *E. coli*. This assessment utilized prior knowledge of the features of “difficult to express” protein families, such as the presence of multiple transmembrane domains, regions of protein disorder and regions of low complexity [16]. The selected proteins were put into expression trials as full-length constructs or as truncated constructs when single transmembrane domains and/or signal peptides needed to be removed to assist soluble protein expression (Table 1).

### Recombinant protein production and preliminary assessment by ELISA

Constructs encoding all or part of the fifteen selected proteins were cloned into a modified pET15bTEV vector which allowed for the production of histidine tagged proteins which could be purified by immobilised metal affinity chromatography followed by tag removal and further purification by size exclusion chromatography. Seven proteins were successfully purified in monomeric form to >95% purity (Figure S1 in Text S1). The remainder were expressed in inclusion bodies and were not pursued further.

**Figure 1:**
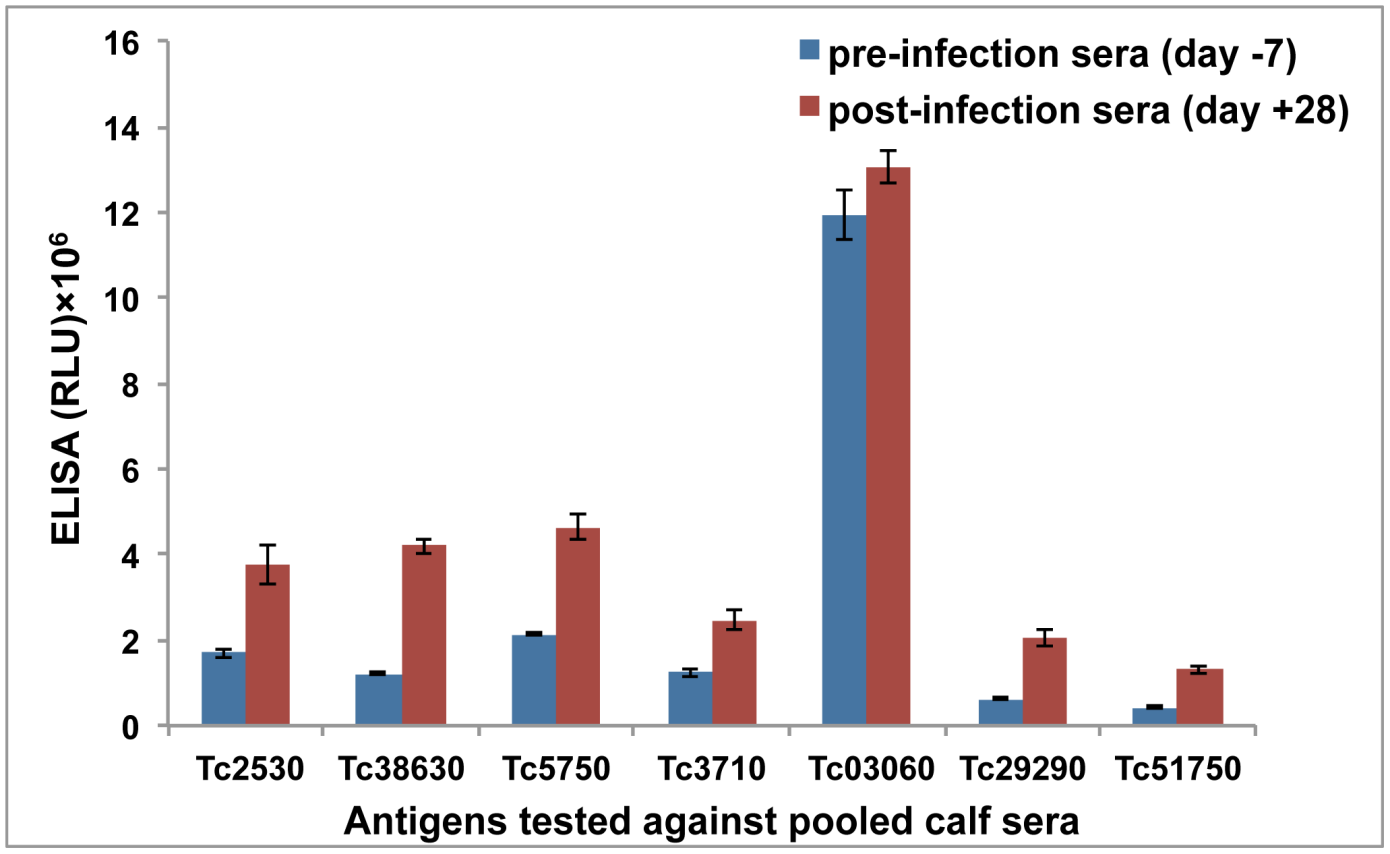
Assessment of the diagnostic potential of recombinant proteins by ELISA. Recombinant proteins were tested by ELISA using pooled sera from the same 40 calves before (day −7; blue bars) and after (day +28; red bars) experimental infection with *T. congolense*. The mean relative light units (RLU) ± SEM from the triplicate measurements are shown.

The successfully expressed and purified monomeric recombinant proteins (Table S1 in Text S1) were tested in ELISA assays for their reactivity to cattle sera. In the initial experiments, we used pooled pre-infection (day −7) and post-infection (day +28) sera (Figure 1). All antigens tested showed significant significant differences in immunoreactivity between pre- and posrt-infection sera (i.e, paired T-test p<0.05).

From this initial assessment, three proteins, all members of the ISG family (Tc38630, Tc29290 and Tc51750), were selected for further investigation as they had post- to pre- infection signal ratios >3. The four other proteins, a member of the PNP_UDP1 superfamily (Tc2530), a tyrosine specific protein phosphatase (Tc5750), an adenosine 5′-monophosphoramidase (Tc3710) and a transferrin receptor-like/PAG-like protein (Tc03060) had relatively poor immunoselectivity ratios and were not progressed further.

### Improved recombinant protein production and assessment of Tc38630, Tc29290 and Tc51750 by ELISA with individual sera

We found that the addition of an N-terminal maltose binding protein (MBP) affinity tag to the *T. congolense* protein constructs generally increased the expression and overall yield of the recombinant proteins by about 20-fold. However, as MBP is an *E. coli*-derived protein it was thought prudent to remove it by proteolytic cleavage before assessing the immunodiagnostic potential of the antigen. This reduced the final yield improvement for the monomeric recombinant protein to about 3-fold. MBP-fusion expressed (with tag subsequently removed) typical yields for Tc38630, Tc29290 and Tc51750 were 2 mg/L, 0.3 mg/L and 4 mg/L respectively. Viable quantities of Tc29290 can only be obtained using the fusion protein.

Purified, MBP-tag cleaved and monomeric (by gel filtration) forms of Tc38630, Tc29290 and Tc51750 were used to create ELISA plates for testing with 40 individual pre- (day −7) and post-infection (day +28) calf sera.

To judge the sensitivity and specificity of each of the three ELISA assays, receiver operating characteristic (ROC) curve analysis was performed. The recombinant Tc38630 protein, with a ROC curve area of 0.954 was clearly superior to Tc29290 and Tc51750 for diagnostic potential (Figure S2 in Text S1).

### Evaluation of the diagnostic potential of Tc38630

**Figure 2:**
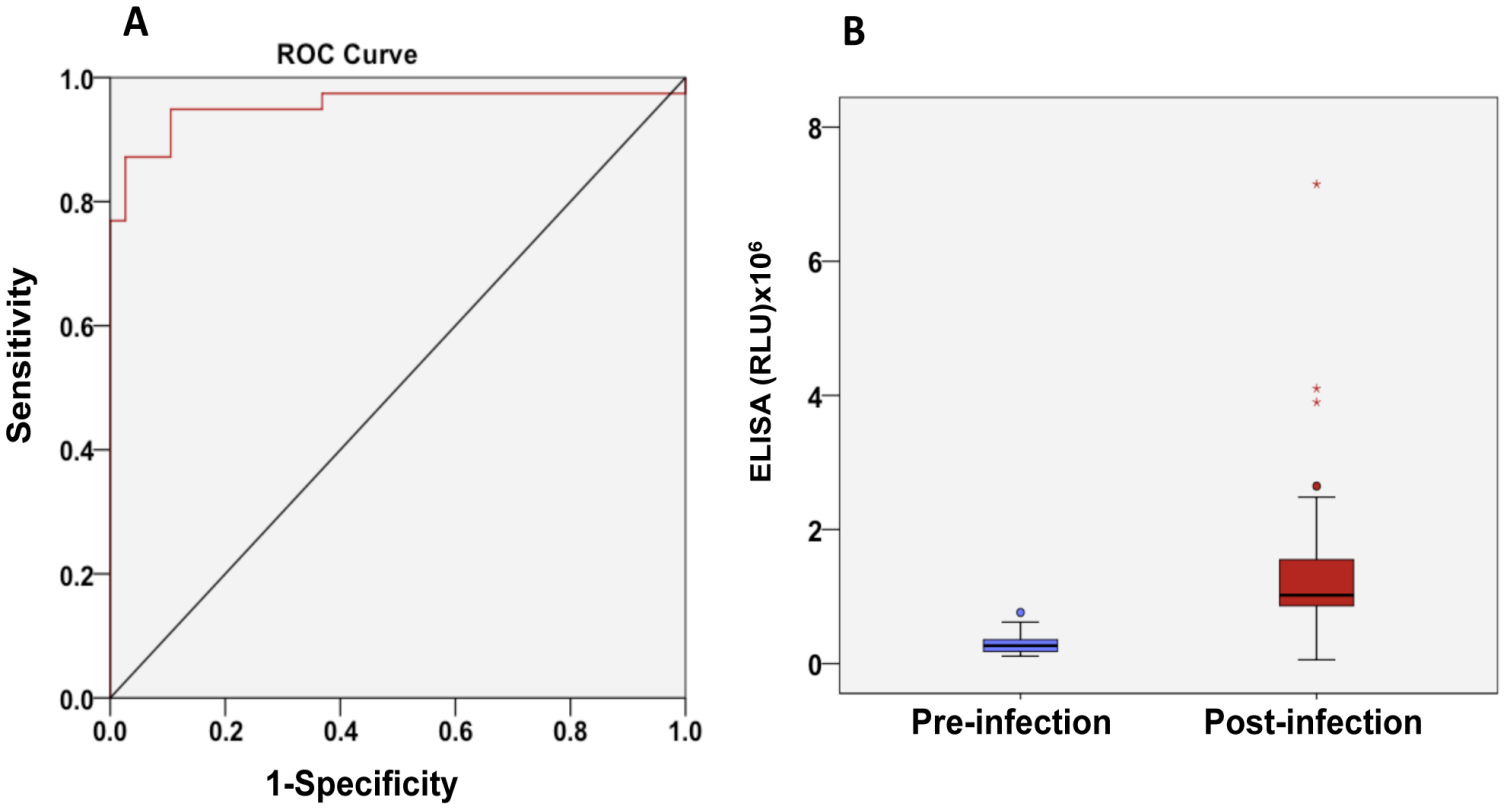
Evaluation of the diagnostic potential of Tc38630 in ELISA assay with pre- and post-infection calf sera. (A) ELISA plates coated with recombinant Tc38630 were tested with 77 blinded and randomised pre (n = 38) and post-infection (n = 39) calf sera. (B) After decoding the results and using a pre-defined cut-off, the data were plotted as ROC curves of specificity against sensitivity (1-specificity). The boxes represent the 25th to 75th percentiles, the horizontal line within the box represents the median value and the whiskers indicate the 10^th^ and 90^th^ percentiles.

Tc38630 was taken forward to be tested with 77 randomised and blinded pre- and post-infection calf sera. Based on the previous data, we selected an initial cut-off of 650000 relative light units (RLU) to predict infected (>650000 RLU) and uninfected (<650000 RLU) calves. Following decoding of the data and ROC curve analysis (Figure 2), we found that Tc38630 ELISA gave a sensitivity of 87.2% and specificity of 97.4%. Exploring alternative cutoff values (Table 2) we can see that the optimal sensitivity of the assay is achieved with a cutoff of 418686 RLU which gave a sensitivity and specificity of 94.9% and 89.5%, respectively. Scatter and box plots indicating the ranges of ELISA readings obtained with pre- and post-infection sera are shown in (Figure 2B and 2C).

**Table 2:** Sensitivity and specificity scores at different RLU cut-offs.

Cutoff>	Sensitivity %	95% CI	Specificity %	95% CI
298493	97.44	86.52% to 99.94%	63.16	45.99% to 78.19%
309600	94.87	82.68% to 99.37%	63.16	45.99% to 78.19%
319127	94.87	82.68% to 99.37%	65.79	48.65% to 80.37%
324493	94.87	82.68% to 99.37%	68.42	51.35% to 82.50%
327533	94.87	82.68% to 99.37%	71.05	54.10% to 84.58%
343107	94.87	82.68% to 99.37%	73.68	56.90% to 86.60%
362373	94.87	82.68% to 99.37%	76.32	59.76% to 88.56%
370027	94.87	82.68% to 99.37%	78.95	62.68% to 90.45%
379000	94.87	82.68% to 99.37%	81.58	65.67% to 92.26%
386747	94.87	82.68% to 99.37%	84.21	68.75% to 93.98%
395113	94.87	82.68% to 99.37%	86.84	71.91% to 95.59%
¶418647	94.87	82.68% to 99.37%	89.47	75.20% to 97.06%
441513	92.31	79.13% to 98.38%	89.47	75.20% to 97.06%
472200	89.74	75.78% to 97.13%	89.47	75.20% to 97.06%
525140	87.18	72.57% to 95.70%	89.47	75.20% to 97.06%
556413	87.18	72.57% to 95.70%	92.11	78.62% to 98.34%
588580	87.18	72.57% to 95.70%	94.74	82.25% to 99.36%
○649520	87.18	72.57% to 95.70%	97.37	86.19% to 99.93%
696160	84.62	69.47% to 94.14%	97.37	86.19% to 99.93%
736407	82.05	66.47% to 92.46%	97.37	86.19% to 99.93%
761707	79.49	63.54% to 90.70%	97.37	86.19% to 99.93%
762747	76.92	60.67% to 88.87%	97.37	86.19% to 99.93%
794293	76.92	60.67% to 88.87%	100	

○ pre-unblinding cut-off; ¶, optimal cut-off deduced after un-blinding the data.

It is worth noting that it has been previously reported that this antigen does not cross react with the common non-pathogenic cattle trypanosome *T. theileri* [11].

### Antibody response to Tc38630 correlates with post-treatment infection status

**Figure 3:**
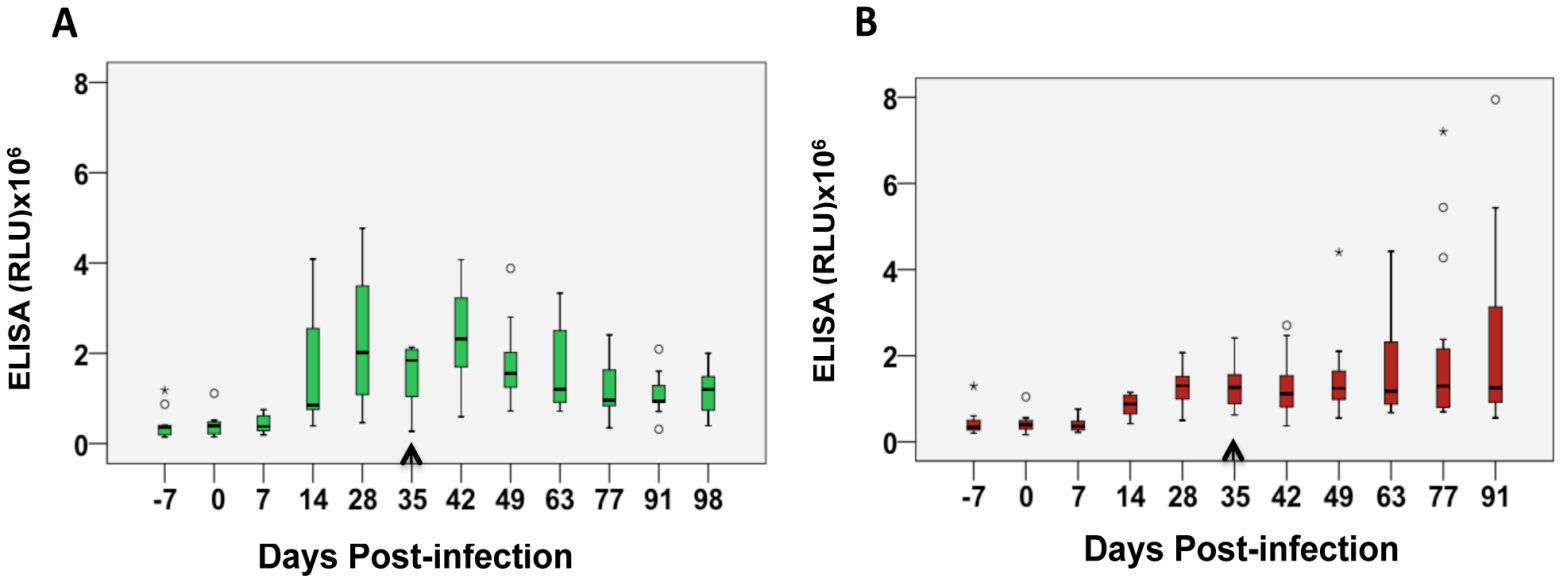
Differential immunoreactivity of sera from animals with aparasitaemia and parasitaemia post-treatment to recombinant Tc38630. ELISA data from cattle sera at different days post-infection are represented as box plots. The boxes represent the 25th to 75th percentiles, the horizontal line within the box represents the median value and the whiskers indicate the 10^th^ and 90^th^ percentiles. The cattle were negative (A) or positive (B) for parasitaemia at the end of treatment. The day of treatment is indicated by a black arrow.

In order to determine if immunoreactivity to Tc38630 might distinguish successful from failed drug-treatment, we first tested sera taken at several time points from 40 calves subjected to experimental infection and drug treatment 35 days later. When the cattle infection status was taken into account (assessed by the presence or absence of parasitemia at day 98) two distinct antibody responses were noted. Thus, relative to immunoreactivity at day 28, persistent and higher immunoreactivity to Tc38630 correlated with presence of parasitaemia at day 98, i.e., failed drug treatment (Figure 3B), while lower immunoreactivity to Tc38630 correlated with aparasitaemia at day 98, i.e., successful drug treatment (Figure 3A).

**Figure 4:**
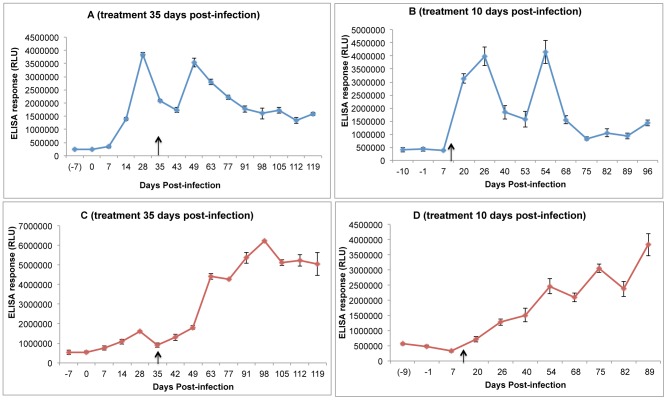
Representative examples of the immunoreactivity of sera to Tc38630 over time. The mean relative light units (RLU) ± SEM from triplicate measurements of immunoreactivity over a time-course are shown. (A) and (B): responses of cattle aparasitemic at the end of the study treated 35 and 10 days post-infection, respectively. (C) and (D): response of cattle with detectable parasitemia at the end of the study treated 35 and 10 days post-infection, respectively. The day of treatment is indicated by a black arrow.

When analysed at the level of individual animal responses, we noticed that the sera from ‘drug-cured’ animals, i.e., those aparasitaemic at the end of the experiment, typically showed a two-wave antibody response to Tc38630 (Figure 4, A and B), whereas animals that remained infected at the end of the study show a constant rise in immunoresponsivenss to Tc38630 (Figure 4, C and D).

Taken together, these data suggest that Tc38630 may have the potential to distinguish successful and failed drug treatments by comparing ELISA titres shortly before treatment with those >50 days later.

## Discussion

In this study, for practical reasons, we have used *T. congolense* cells propagated in mice to generate bloodstream form parasite lysates for affinity chromatography on immobilized bovine IgG matrices. Since it is conceivable that host animal environment may influence parasite gene expression, we must acknowledge that there may consequently be limitations in the list of specifically-recognized antigens (Table S1 in Text S1). Nevertheless, three proteins belonging to the invariant surface glycoprotein (ISG) family (Tc38630, Tc45510 and Tc29290) were successfully identified as potential diagnostic antigens by proteomic selection followed by assessment of likely ease of expression in *E. coli* followed by protein expression trials. The best performing of these, Tc38630, was also recently reported as a potential diagnostic antigen for *T. congolense* infections using experimental mouse sera [11]. Although annotated as hypothetical in the genome database, Tc38630 has been previously identified by quantitative proteomics as being most abundant on the metacyclic and bloodstream trypomastigote forms of *T. congolense* [17].

The ISGs have, thus far, only been characterised in *T. brucei*, where ISG65 and ISG75 have been shown to be moderately abundant (50000 to 70000 copies per cell) type-1 integral membrane cell surface glycoproteins [18]. While the physiological function of the ISGs is unclear, they are known to be endocytosed and recycled [19] and ISG75 has been implicated as the principal receptor for the uptake of the drug suramin [20]. The surface location, reasonable abundance and lack of similarity with any mammalian proteins most likely all contribute to the immunogenicity of ISGs in animal infections. Indeed, ISGs have been proposed and/or used as diagnostic antigens for human or animal infections with *T. brucei gambiense* [7], [21], *T. evansi* [22] and *T. congolense* [11]. Nevertheless, choosing which of the many (49 in the case of *T. congolense*) ISG protein sequences in each trypanosome species [23] to select for protein expression and ELISA trials is a daunting task without the prioritisation provided by the proteomic approach taken in [7] for *T. brucei* and in this paper for *T. congolense*. Other selection criteria can lead to disappointing results; for example, the *T. congolense* ISG with the greatest similarity to that selected for the diagnosis of human *T. brucei gambiense* infections proved to be poor for the diagnosis of *T. congolense* infections in cattle (unpublished data). Further, although there is a lack of ISG sequence similarity between trypanosome species (typically <10% sequence identity and <20% sequence similarity) [23] it would be premature to assume that immunoreactivity to Tc38630 is specific for *T. congolense* bovine infections over *T. brucei* and *T. vivax* infections, and this issue awaits analysis.

In summary, we have identified a member of the invariant surface glycoprotein family (Tc38630) as a diagnostic antigen for the detection of *T. congolense* infection in cattle. It is known that serum antibody persistence in Trypanosome infected cattle after curative treatment, or self-cure, is on average 3–4 months [24] and can be up to 13 months [25]. At the point of care, an ideal diagnostic antigen would be able to indicate active cattle infections with high sensitivity and specificity and be able to accurately track drug efficacy. The latter requires that immunoreactivity to the antigen is maintained in continuing infections and significantly reduces following cure. The data provided here suggests that this may well be the case for the recombinant Tc38630 protein construct. Therefore, further development of this antigen into lateral flow devices may be warranted. Nevertheless, it must be acknowledged that antigen, as opposed to antibody, detection tests are undoubtedly better for monitoring pathogen burden and drug efficacy. Unfortunately, antigen detection systems for AAT are currently lacking.

## Supporting Information

Text S1 **Figure S1. SDS-PAGE with Coomassie blue staining of purified recombinant proteins.** The names of the expressed proteins appear above each respective gel lane. The bands indicated by arrow heads are: A, Tc38630; B, degradation product of Tc38630; C, *E. coli* Ef-Tu (co-purifying contaminant); D, Tc29290. These identities were confirmed by tryptic digestion and mass spectrometry. **Figure S2. Assessment of Tc38630, Tc29290 and Tc51750 ELISA assays with pre- and post-infection calf sera.** (A) ELISA plates coated with the three recombinant proteins were tested with pre-infection (day −7; n = 40) and post-infection (day +28; n = 40) calf sera. The data were plotted on RoC curves of specificity against selectivity (1-specificity). The output statistics show a sensitivity and specificity of 90% and 94.2% for Tc38630, 80% and 67.3% for Tc29290 and 82.5% and 67.3% for Tc51750, respectively. **Table S1. Antigens selectively recognised by** ***T. congolense*** **infection IgG.** The antigens are ordered by infection : control LC-MS/MS intensity and coloured coded according to their by LC-MS/MS intensities: black bold >1000, black >60, grey <60. Only antigens uniquely bound by infection IgG to or with high intensity (>1000) and an infection : control ratio >100 were assigned identities. Those that failed to yield soluble protein in expression and purification trials are marked with ▪. Those selected but untested in expression trials are marked with white rectangle (vectors available on request) and those purified successfully are marked white rectangle. **Table S2. Amino acid sequences of the seven antigen domains successfully expressed in** ***E. coli*** **.** Differences in sequence to those in the TriTrypDB are highlighted in red, these are probably due to strain variation.
(DOC)
