# Supplementary material for: Proteomic Selection of Immunodiagnostic Antigens for Trypanosoma congolense
Source: PLoS Negl Trop Dis. 2014 Jun 12;8(6):e2936. doi: 10.1371/journal.pntd.0002936 (PMC4055490; doi:10.1371/journal.pntd.0002936)
Supplement: Text S1 — Figure S1. SDS-PAGE with Coomassie blue staining of purified recombinant proteins. The names of the expressed proteins appear above each respective gel lane. The bands indicated by arrow heads are: A, Tc38630; B, degradation product of Tc38630; C, E. coli Ef-Tu (co-purifying contaminant); D, Tc29290. These identities were confirmed by tryptic digestion and mass spectrometry. Figure S2. Assessment of Tc38630, Tc29290 and Tc51750 ELISA assays with pre- and post-infection calf sera. (A) ELISA plates coated with the three recombinant proteins were tested with pre-infection (day −7; n = 40) and post-infection (day +28; n = 40) calf sera. The data were plotted on RoC curves of specificity against selectivity (1-specificity). The output statistics show a sensitivity and specificity of 90% and 94.2% for Tc38630, 80% and 67.3% for Tc29290 and 82.5% and 67.3% for Tc51750, respectively. Table S1. Antigens selectively recognised by T. congolense infection IgG. The antigens are ordered by infection : control LC-MS/MS intensity and coloured coded according to their by LC-MS/MS intensities: black bold >1000, black >60, grey <60. Only antigens uniquely bound by infection IgG to or with high intensity (>1000) and an infection : control ratio >100 were assigned identities. Those that failed to yield soluble protein in expression and purification trials are marked with ▪. Those selected but untested in expression trials are marked with white rectangle (vectors available on request) and those purified successfully are marked white rectangle. Table S2. Amino acid sequences of the seven antigen domains successfully expressed in E. coli . Differences in sequence to those in the TriTrypDB are highlighted in red, these are probably due to strain variation. (DOC) [file pntd.0002936.s001.doc]

**Text S1**

**Figure S1.** **SDS-PAGE with Coomassie blue staining of purified recombinant proteins.** The names of the expressed proteins appear above each respective gel lane. The bands indicated by arrow heads are: A, Tc38630; B, degradation product of Tc38630; C, *E. coli* Ef-Tu (co-purifying contaminant); D, Tc29290. These identities were confirmed by tryptic digestion and mass spectrometry.


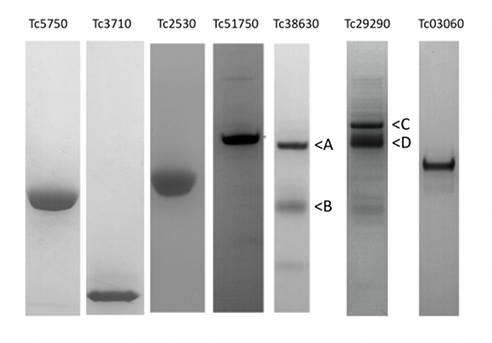


**Figure S2. Assessment of Tc38630, Tc29290 and Tc51750 ELISA assays with pre- and post-infection calf sera.** (A) ELISA plates coated with the three recombinant proteins were tested with pre-infection (day -7; n=40) and post-infection (day +28; n=40) calf sera. The data were plotted on RoC curves of specificity against selectivity (1-specificity). The output statistics show a sensitivity and specificity of 90% and 94.2% for Tc38630, 80% and 67.3% for Tc29290 and 82.5% and 67.3% for Tc51750, respectively.

|  | **Cutoff >** | **Sensitivity %** | **95% CI** | **Specificity %** | **95% CI** |
| --- | --- | --- | --- | --- | --- |
| **Tc29290** | 1062000 | 80 | 64.35% to 90.95% | 67.31 | 52.89% to 79.67% |
| **Tc51750** | 1416000 | 82.5 | 67.22% to 92.66% | 67.31 | 52.89% to 79.67% |
| **Tc38630** | 691800 | 90 | 76.34% to 97.21% | 94.23 | 84.05% to 98.79% |


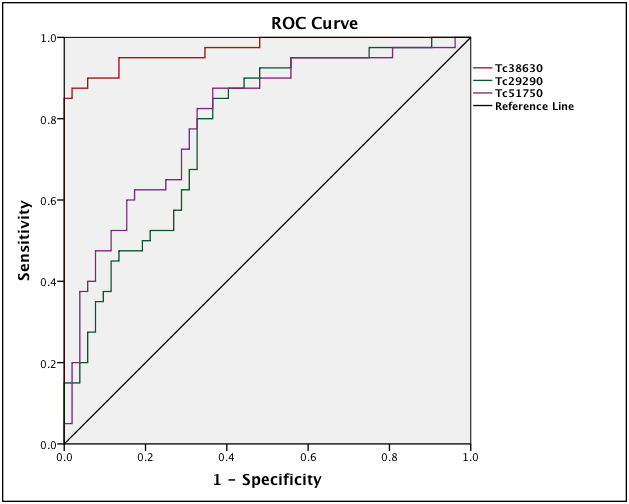


**Table S1. Antigens selectively recognised by *T. congolense* infection IgG.** The antigens are ordered by infection : control LC-MS/MS intensity and . coloured coded according to their by LC-MS/MS intensities: black bold >1000, black >60, grey <60. Only antigens uniquely bound by infection IgG to or with high intensity (>1000) and an infection : control ratio >100 were assigned identities. Those that failed to yield soluble protein in expression and purification trials are marked with ◼. Those selected but untested in expression trials are marked with ✜ (vectors available on request) and those purified successfully are marked with ✔.

| GeneDB code | LC MS/MS intensity x 10^6^ | | infected:control  ratio | Putative name/protein family |
| --- | --- | --- | --- | --- |
|  | Control | infected |  |  |
| TcIL3000.0.47660 | 0 | 94.32 | unique | none |
| TcIL3000.0.47360 | 0 | 79.55 | unique | pha03247, splicing factor/polymerase |
| TcIL3000.0.17670 | 0 | 78.93 | unique | VSG, atypical, putative |
| TcIL3000.0.54810 | 0 | 40.16 | unique | none |
| TcIL3000.0.02080 | 0 | 28.26 | unique | VSG, atypical, putative |
| TcIL3000.8.2260 | 0 | 26.90 | unique | tRNA_lig_CPD |
| TcIL3000.0.22930 | 0 | 26.71 | unique | ESAG6/7 |
| TcIL3000.0.12490 | 0 | 23.17 | unique | ESAG2 |
| TcIL3000.0.55840 | 0 | 16.23 | unique | C1A peptidase |
| TcIL3000.0.12660 | 0 | 15.80 | unique | VSG |
| TcIL3000.0.54690 | 0 | 15.62 | unique | ESAG2 |
| TcIL3000.0.33860 | 0 | 14.20 | unique | trans-sialidase, putative |
| ◼ TcIL3000.0.46780 | 0 | 14.17 | unique | ESAG6/7 |
| TcIL3000.0.46270 | 0 | 14.03 | unique | VSG putative |
| TcIL3000.0.55740 | 0 | 13.97 | unique | None |
| TcIL3000.0.01140 | 0 | 13.72 | unique | Trans-sialidase |
| ◼ TcIL3000.0.44290 | 0 | 13.71 | unique | ESAG2 |
| TcIL3000.0.54680 | 0 | 13.49 | unique | VSG/ESAG |
| TcIL3000.0.44210 | 0 | 12.94 | unique | cathepsin L-like protease |
| TcIL3000.8.1810 | 0 | 10.42 | unique | Golgi/lysosome glycoprotein 1, putative |
| TcIL3000.0.34020 | 0 | 10.39 | unique | procyclin associated gene |
| TcIL3000.0.47860 | 0 | 10.02 | unique | ESAG2 |
| TcIL3000.0.50100 | 0 | 8.24 | unique | VSG putative |
| TcIL3000.6.3420 | 0 | 7.92 | unique | None |
| TcIL3000.0.42220 | 0 | 7.32 | unique | ISG65 |
| TcIL3000.0.41550 | 0 | 7.13 | unique | VSG_b  ESAG2 |
| TcIL3000.0.34030 | 0 | 6.11 | unique | procyclin associated gene |
| TcIL3000.0.37630 | 0 | 5.96 | unique | VSG_b |
| TcIL3000.0.05210 | 0 | 5.81 | unique | ESAG2 |
| TcIL3000.0.41560 | 0 | 5.43 | unique | ESAG2 |
| TcIL3000.0.14680 | 0 | 5.33 | unique | VSG putative |
| TcIL3000.0.56080 | 0 | 4.99 | unique | cysteine peptidase C |
| TcIL3000.0.06710 | 0 | 4.37 | unique | VSG_b |
| ◼ TcIL3000.0.43930 | 0 | 4.34 | unique | ESAG2 |
| TcIL3000.0.07360 | 0 | 4.15 | unique | VSG/ESAG |
| TcIL3000.0.49100 | 0 | 4.12 | unique | VSG/ESAG |
| TcIL3000.10.2050 | 0 | 4.11 | unique | major surface protease gp63 |
| TcIL3000.0.32750 | 0 | 3.64 | unique | ESAG6 |
| TcIL3000.6.1210 | 0 | 3.61 | unique | 3'-nucleotidase/nuclease, putative |
| TcIL3000.0.01460 | 0 | 3.52 | unique | ESAG6/7 |
| TcIL3000.0.39320 | 0 | 3.17 | unique | ESAG2 |
| TcIL3000.0.38220 | 0 | 3.03 | unique | VSG put |
| ✔ TcIL3000.0.35150 | 0 | 2.80 | unique | ISG65 |
| TcIL3000.0.17210 | 0 | 2.75 | unique | ISG65 |
| TcIL3000.6.1720 | 0 | 2.50 | unique | histidyl-tRNA synthetase, putative |
| TcIL3000.0.44760 | 0 | 2.43 | unique | VSG_b |
| TcIL3000.0.55700 | 0 | 2.29 | unique | VSG putative |
| TcIL3000.4.120 | 0 | 2.02 | unique | 20s proteasome β7 subunit |
| TcIL3000.0.57350 | 0 | 2.01 | unique | ESAG6/7 |
| TcIL3000.10.13370 | 0 | 1.91 | unique | FKBP-type peptidyl-prolyl cis-trans isomerase, putative |
| ◼ TcIL3000.6.2830 | 0 | 1.89 | unique | IFT  IFT20 |
| ✜ TcIL3000.0.27180 | 0 | 1.85 | unique | 1,2-Dihydroxy-3-keto-5-methylthiopentene dioxygenase, putative |
| TcIL3000.0.38010 | 0 | 1.70 | unique | ESAG2 |
| TcIL3000.0.37980 | 0 | 1.70 | unique | ESAG6/7 |
| TcIL3000.11.3140 | 0 | 1.66 | unique | ubiquitin-conjugating enzyme-like protein |
| TcIL3000.9.3050 | 0 | 1.55 | unique | fructose-1,6-bisphosphate, cytosolic, putative |
| TcIL3000.7.2100 | 0 | 1.46 | unique | none |
| TcIL3000.7.940 | 0 | 1.44 | unique | 10 kDa heat shock protein, putative |
| TcIL3000.9.1350 | 0 | 1.37 | unique | fatty acyl CoA synthetase 4, putative |
| TcIL3000.6.120 | 0 | 1.32 | unique | CCR4 associated factor |
| TcIL3000.0.60660 | 0 | 1.29 | unique | none |
| TcIL3000.0.12460 | 0 | 1.23 | unique | ISG65 |
| TcIL3000.0.22080 | 0 | 1.22 | unique | ISG65 |
| TcIL3000.5.1060 | 0 | 1.22 | unique | armet |
| TcIL3000.0.59860 | 0 | 1.19 | unique | VSG_b |
| TcIL3000.0.20590 | 0 | 1.09 | unique | 60S ribosomal protein L13a |
| TcIL3000.0.12850 | 0 | 1.07 | unique | VSG putative |
| TcIL3000.10.12630 | 0 | 1.03 | unique | clatherin |
| TcIL3000.0.18780 | 0 | 0.91 | unique | ISG75 |
| TcIL3000.0.15580 | 0 | 0.90 | unique | putative kinesin K39 |
| TcIL3000.0.09500 | 0 | 0.82 | unique | trans-sialidase, putative |
| TcIL3000.0.47970 | 0 | 0.79 | unique | VSG_b |
| TcIL3000.10.2810 | 0 | 0.76 | unique | 60S acidic ribosomal protein P2, putative |
| TcIL3000.0.12130 | 0 | 0.70 | unique | VSG_b |
| TcIL3000.0.15690 | 0 | 0.66 | unique | ISG75 |
| TcIL3000.0.05190 | 0 | 0.66 | unique | ESAG2 |
| TcIL3000.0.41700 | 0 | 0.63 | unique | VSG_b |
| TcIL3000.2.1300 | 0 | 0.61 | unique | none |
| TcIL3000.10.3920 | 0 | 0.61 | unique | proteasome beta 2 subunit |
| TcIL3000.10.2160 | 0 | 0.50 | unique | adenylate kinase, putative |
| TcIL3000.10.5120 | 0 | 0.46 | unique | clatherin |
| TcIL3000.7.3960 | 0 | 0.45 | unique | proteasome β6 subunit |
| TcIL3000.5.2920 | 0 | 0.45 | unique | mitochondrial DNA polymerase beta |
| ◼ TcIL3000.0.59160 | 0 | 0.44 | unique | VSG_b |
| TcIL3000.9.5010 | 0 | 0.43 | unique | transport protein particle (TRAPP) subunit |
| TcIL3000.8.740 | 0 | 0.43 | unique | Qa-SNARE |
| TcIL3000.0.22370 | 0 | 0.40 | unique | NADH-dependent fumarate reductase-like protein |
| TcIL3000.9.5750 | 0 | 0.37 | unique | importin/ transportin |
| TcIL3000.0.55710 | 0 | 0.36 | unique | VSG, putative |
| TcIL3000.0.19630 | 0 | 0.36 | unique | peroxisomal enoyl-CoA hydratase |
| TcIL3000.8.6800 | 0 | 0.35 | unique | TRAPP |
| TcIL3000.8.240 | 0 | 0.30 | unique | PPIase, putative |
| TcIL3000.9.3720 | 0 | 0.27 | unique | VSG |
| TcIL3000.0.50050 | 0 | 0.25 | unique | VSG |
| TcIL3000.9.5370 | 0 | 0.20 | unique | transport protein particle (TRAPP) subunit |
| TcIL3000.0.54430 | 0 | 0.16 | unique | ESAG2 |
| TcIL3000.8.1660 | 0 | 0.13 | unique | roblc1 |
| TcIL3000.0.34210 | 0 | 0.12 | unique | VSG |
| **TcIL3000.0.28450** | **1.23** | **2445.40** | **1987** | cysteine peptidase C |
| **TcIL3000.0.27910** | **3.02** | **4654.50** | **1539** | procyclin-associated gene 2 (PAG2) maybe an ESAG6 |
| **TcIL3000.0.55820** | **2.95** | **3907.90** | **1324** | cathepsin L-like protease |
| **TcIL3000.0.14840** | **18.83** | **21004.00** | **1115** | procyclin-associated gene 2 maybe ESAG6/7 |
| TcIL3000.0.00850 | 1.27 | 807.76 | 636 |  |
| **TcIL3000.0.44390** | **10.93** | **6002.50** | **549** | PAG2 possibly ESAG6 |
| **✔ TcIL3000.0.38630** | **4.13** | **2226.90** | **540** | ISG |
| **◼ TcIL3000.10.560** | **7.01** | **3459.90** | **494** | serine carboxypeptidase III precursor |
| **TcIL3000.0.26770** | **28.48** | **13206.00** | **464** | cathepsin L-like protease |
| **TcIL3000.0.31720** | **36.24** | **12811.00** | **354** | cathepsin L-like protease |
| **TcIL3000.0.41540** | **4.06** | **1427.80** | **352** | VSG |
| **◼ TcIL3000.10.860** | **91.83** | **30678.00** | **334** | serine carboxypeptidase III precursor |
| **TcIL3000.0.19600** | **5.24** | **1568.20** | **299** | VSG |
| TcIL3000.0.55830 | 1.85 | 546.02 | 295 |  |
| **TcIL3000.0.18880** | **7.54** | **2115.80** | **280** | cathepsin L-like protease (CPB) |
| **TcIL3000.0.37070** | **32.40** | **9009.90** | **278** | lysosomal/endosomal membrane protein p67 |
| **◼ TcIL3000.2.1660** | **8.28** | **2250.20** | **272** | VSG lipase |
| **TcIL3000.0.03600** | **48.84** | **13053.00** | **267** | cysteine peptidase C (CPC) |
| **✔ TcIL3000.0.03060** | **5.73** | **1197.90** | **209** | PAG2 maybe ESAG 6 |
| **TcIL3000.6.60** | **38.90** | **6321.00** | **162** | cysteine peptidase C (CPC) |
| **✔ TcIL3000.0.51750** | **19.30** | **2738.50** | **142** | ISG |
| TcIL3000.0.29480 | 2.03 | 235.57 | 116 |  |
| TcIL3000.0.03850 | 0.65 | 42.49 | 66 |  |
| TcIL3000.0.23350 | 9.62 | 579.20 | 60 |  |
| TcIL3000.10.12030 | 2.75 | 152.00 | 55 |  |
| TcIL3000.0.54660 | 0.99 | 52.62 | 53 |  |
| TcIL3000.0.29300 | 1.80 | 81.70 | 45 |  |
| TcIL3000.0.41470 | 1.35 | 56.64 | 42 |  |
| TcIL3000.0.45510 | 2.31 | 74.92 | 32 |  |
| TcIL3000.0.14700 | 29.01 | 922.00 | **32** |  |
| TcIL3000.10.250 | 3.63 | 112.27 | 31 |  |
| TcIL3000.11.3680 | 10.58 | 299.75 | 28 |  |
| TcIL3000.0.00360 | 1.16 | 32.11 | 28 |  |
| TcIL3000.3.1460 | 1.32 | 34.60 | 26 |  |
| TcIL3000.0.52730 | 1.36 | 32.73 | 24 |  |
| TcIL3000.10.8840 | 3.79 | 88.25 | 23 |  |
| TcIL3000.11.890 | 1.21 | 27.68 | 23 |  |
| TcIL3000.11.9490 | 12.18 | 274.34 | 23 |  |
| TcIL3000.10.5210 | 4.88 | 109.73 | 23 |  |
| TcIL3000.10.220 | 29.44 | 629.40 | 21 |  |
| TcIL3000.8.2290 | 0.78 | 15.53 | 20 |  |
| REV__TcIL3000.9.4670 | 0.45 | 8.31 | 18 |  |
| TcIL3000.10.11990 | 0.51 | 9.02 | 18 |  |
| TcIL3000.0.56270 | 13.42 | 232.50 | 17 |  |
| TcIL3000.0.34540 | 1.37 | 23.29 | 17 |  |
| TcIL3000.3.3160 | 2.24 | 38.16 | 17 |  |
| TcIL3000.10.9550 | 3.61 | 61.28 | 17 |  |
| TcIL3000.9.1930 | 0.65 | 10.74 | 17 |  |
| TcIL3000.4.1900 | 0.93 | 15.42 | 17 |  |
| TcIL3000.10.13840 | 0.84 | 13.83 | 16 |  |
| TcIL3000.0.42510 | 12.96 | 213.54 | 16 |  |
| TcIL3000.8.5570 | 4.95 | 80.65 | 16 |  |
| TcIL3000.0.00860 | 0.75 | 12.08 | 16 |  |
| TcIL3000.6.820 | 2.06 | 32.19 | 16 |  |
| TcIL3000.10.7090 | 0.30 | 4.64 | 16 |  |
| TcIL3000.0.29070 | 0.73 | 11.35 | 15 |  |
| TcIL3000.5.1320 | 1.62 | 24.67 | 15 |  |
| TcIL3000.0.22710 | 12.24 | 185.65 | 15 |  |
| TcIL3000.11.15910 | 1.01 | 15.04 | 15 |  |
| TcIL3000.10.2660 | 0.88 | 12.87 | 15 |  |
| TcIL3000.1.360 | 1.15 | 16.46 | 14 |  |
| TcIL3000.0.38940 | 1.68 | 23.80 | 14 |  |
| TcIL3000.9.1910 | 0.87 | 12.10 | 14 |  |
| TcIL3000.10.100 | 4.91 | 67.88 | 14 |  |
| TcIL3000.0.60550 | 2.94 | 39.91 | 14 |  |
| TcIL3000.10.1960 | 0.40 | 5.43 | 13 |  |
| TcIL3000.6.3740 | 2.19 | 28.43 | 13 |  |
| TcIL3000.0.00060 | 1.42 | 18.00 | 13 |  |
| TcIL3000.11.8120 | 0.56 | 6.99 | 12 |  |
| TcIL3000.0.11300 | 0.46 | 5.65 | 12 |  |
| TcIL3000.10.4760 | 7.28 | 87.71 | 12 |  |
| TcIL3000.10.12610 | 0.93 | 11.02 | 12 |  |
| TcIL3000.0.17680 | 7.08 | 83.63 | 12 |  |
| TcIL3000.10.12040 | 8.21 | 91.34 | 11 |  |
| TcIL3000.0.55170 | 5.01 | 55.61 | 11 |  |
| TcIL3000.7.3730 | 41.26 | 444.05 | 11 |  |
| TcIL3000.0.20480 | 1.50 | 16.02 | 11 |  |
| TcIL3000.0.17880 | 3.10 | 32.87 | 11 |  |
| TcIL3000.5.3990 | 2.04 | 21.29 | 10 |  |
| TcIL3000.6.3920 | 0.83 | 8.63 | 10 |  |
| TcIL3000.11.660 | 2.40 | 24.78 | 10 |  |
| TcIL3000.10.11960 | 0.77 | 7.89 | 10 |  |
| TcIL3000.4.1670 | 4.90 | 49.72 | 10 |  |

**Table S2. Amino acid sequences of the seven antigen domains successfully expressed in *E. coli.*** Differences in sequence to those in the TriTrypDB are highlighted in red, these are probably due to strain variation.

>TcIL3000.0.38630| Residues 30-355

AQESNATEEDVESSSVPTKTDEEKVFIRNVTCEADIYASRALCAFPRLMKEVEQERHVAGDNIKGDVENKAQKAHDAAEALRGAISLAKDTLNDPEGKLVKVADNLEEISKEDLGLVQTMVDEANKASEEVGELYKEASKRTPGEGKCSGESGIKSFYGCGDLKGIRFSNYSDGCGPLGMDNLLGHVGPLACKKTPNCHGALRNGLLLTLYVREWDYTRPLVKDECTDKDEWLKDIEDVGRAMKDLGNKINDFNATLSKIDKYIDVVVKITEMVKDGKSYEDILKAVEGMKSASDGSSSMLKTGSEEDPFNISELMNGRETFWGKYS

>TcIL3000.9.2530| Full length

MRRGRVRTESTIQRFSKTPIGEGFDLWDRD**Y**LLGAIRLFL**F**KAETAPPFQVGPCMDATAEILMQMEEIEDAGEQFTAAAEKYSVTQQPQLSKLMKIKALECSEGPTVALAELTKFLDEEISSASAATFSPPVVRACAYQAELLLKTANGQTPEVVEKAIEVAKLSCNHQWDRVHRGYIALGDALTAGGRRVDAVKAFKDALEVSPNCLAAIERHISVLKDLIAEGGDDARIKQLRTEMLGLLSRAIDLHPRPTLIREKAFLLSETEGDEKALEFLEPLICNPPPQEADATNKCGGETIATLLKAKAAILADGGKLQEALTVAELALKESPGDEEAMSIVAELRESM

>TcIL3000.10.5750|Full length

MSTTDKFPMAELSTQLQLARMQREFAQLQKQENPRTIDFTTSLCNRHKNRYLDILANEETIYPPVPKGAENDRGHCCYINGNFVDLDLPHKFVACQAPVPQGMPDFLETLAHGKVNLVLMLTKLREGGVLKADRYWPEEGEEELSFPLPEGGVVTVRMDPEVPYEVDSTLDITRRKLIINMPGK**P**PHQLLQVQYTGWPDHGVPESAAAFDALLSVIKDSTTTSPILIHCSAGIGRTGTLIGAYAGITHIERDTLSDTTVYSIVAAMKRKRLGMVQRLEQYAVIYITVLSRLGVDISGLVSTLNIKAGPSAA

>TcIL3000.7.371|Full length

MSSCIFCRIVEGSIPCHKVAETAKVLAFMDINPISRGHLLVIPKAHAEFLHEVEPSTAAEVGETLAKITRAIVSEEGVGKQYNILQNNGKLAHQEVPHVHFHIIPRRNTEEGLGITWNSQPTDHPALAEQAEKYRQALEKVL

>TcIL3000.0.29290| Residues 26-362

AGNSNGVCRLNENAAGLLCTIAKLVEKAKNITENHDYKDIDETWGYVALHKEVVDHRVKNLPDIIETAKAKGTLTDKDAEHLTTLYLDAHNKNTQQHNKSKAAMDAHNKTHEDAKNSTALALGEGYVTGNCNMVSSLLGILQCYVKGEQPHSNLNVETICKEKNYNLDESQNTLLTNCNKIGNHKTYCNGTGAALKVALEKWNGMDKKKAAENGNCEVKKDWEERTKKAQEHMSRLDEHVQIIHDAKLLTTAYFAIVDKIKTGVENGKPMKVIVANAREAGQKGAKVVVGKLSIHTENDTHNTTKPLLEEEEVNVNVQLDGLKFDEDENGPAHSKES

>TcIL3000.0.51750| Residues 36- 364

MLDRFGASALCTMKVYCDRIVSDTAEYLVEEIGYQLYLIKSDIHSINHYTGEINRYFGDLPQDQQEKVMKACEVALNITKARREIAEAAKKKAEDLVLNATLAANLTLGEIGEDEDGNATVDNTTGLARVINWHCGYSKVENETEPPSTSCHVVGKRKRFSGGFRNTISCQRLEKSVSYKNATAENMRRALMEWNVVKPMPKENGKICQDTDDHTNHSCTVWEGWVADYKKTTDLMKKLGGAERRARRARLQGEVEVIGLYKIYQALRAGEESDVIELLLEEAEEKRMLANEAELVSGVLVTRGSDKMSLERDKDLKLLKQARTETPKG

>TcIL3000.0.03060| Residues 28-374

TEEAKTFLRLSKARSICNASRTMKGVHSYVSRKITEYQKKYDEMDMIRDVARLKVLMKVNLDAKCGREATFLLYVQEGMIAFREALEKLRAAGVRAVASAGIAAGRLDEFMSVFKQAHGKNNDPRNCAGKIGSVVDGMKLMLKECYRGATIEDEFHSVADVEAEFGVKDLELEEALKKHLTANNEGTGASNDPTICNLGIIDNNTKTYVKDETPKEIKWGDGVLLLKDGSGGGDKYKWAESPTTAIPVIKSAISDFSDFKKAVTDVERCYAGLRDDWTDSRLKEADMQRILASLKYNKFNTSEAVSFHVLSSLQTDENNTNVLFPEWNREKVAGKIWKRNWLFVMAK
